# Short-term mortality after opioid initiation among opioid-naïve and non-naïve patients with dementia: a retrospective cohort study

**DOI:** 10.1186/s12916-025-04172-1

**Published:** 2025-06-09

**Authors:** Yeon-Mi Hwang, Jennifer M. Hah, Jennifer E. Bramen, Jennifer J. Hadlock, Tina Hernandez-Boussard

**Affiliations:** 1 Department of Medicine, School of Medicine, Stanford University, Palo Alto, CA USA; 2 Department of Anesthesiology, Perioperative and Pain Medicine, School of Medicine, Stanford University, Stanford, CA USA; 3 Pacific Neuroscience Institute Foundation, Santa Monica, CA USA; 4 Pacific Brain Health Center, Providence Saint John’s Health Center, Santa Monica, CA USA; 5 Institute for Systems Biology, Seattle, WA USA; 6 Department of Biomedical Informatics and Education, School of Medicine, University of Washington, Seattle, WA USA

**Keywords:** Dementia, Opioid safety, Mortality risk, Pain management, Cognitive impairment, Large language model, Healthcare system

## Abstract

**Background:**

In the ongoing opioid epidemic, the mortality risk of opioid initiation in patients with dementia or mild cognitive impairment (MCI) remains understudied despite their vulnerability. This study evaluates mortality risks associated with opioid exposure in patients diagnosed with dementia or MCI by comparing outcomes between the initiation and continuation groups.

**Methods:**

We conducted a retrospective cohort study using data from a Northern California academic healthcare system (Stanford Health Care Alliance; 2015/01/01–2024/07/31), including 27,757 patients aged 50–100 with dementia or MCI. Of these, 14,105 received opioids after diagnosis and were classified as initiation (opioid-naïve; *n*=9443) or continuation (non-naïve; *n*=4662) groups. Cox regression assessed 14-day mortality risk. Aalen’s additive model examined time-varying impact up to 180 days. Potential causes of death were extracted from clinical notes using GPT-3.5-Turbo. We also analyzed an independent community healthcare system cohort (Providence Health & Service; *n*=208,306) from western US states (2015/01/01–2023/05/31) as a replication cohort.

**Results:**

In the primary cohort, 4.1% (572/14,105) of patients died within 14 days of opioid exposure. The initiation group had a significantly higher 14-day mortality risk than the continuation group (adjusted hazard ratio (aHR), 2.00 (1.59–2.52); *P*<0.0001). The replication cohort had a 14-day mortality rate of 6.2% (7022/113,343) with a smaller difference between the initiation (*n*=77,168) and continuation (*n*=36,175) groups (aHR 1.22 (1.16–1.30); *P*<0.0001). In both cohorts, elevated risk stabilized after day 30. In the primary cohort, respiratory conditions (62% vs. 48%, *P*<0.1), particularly pneumonia (38% vs. 19%, *P*<0.05), were more prevalent among the initiation group who died early.

**Conclusions:**

Starting opioids in patients with dementia or MCI is associated with elevated short-term mortality risks, with the initiation group having twice the 14-day mortality risk in academic settings and a smaller but significant increase in community healthcare systems. The first 30 days after initiation represent a critical risk window, likely due to a lack of tolerance to opioid adverse effects. These findings underscore the need for cautious initiation, tailored follow-up protocols accounting for healthcare setting characteristics, and close monitoring during the first month in this vulnerable population.

**Supplementary Information:**

The online version contains supplementary material available at 10.1186/s12916-025-04172-1.

## Background

Pain is a common experience among patients with dementia [1–3], frequently arising from age-related comorbidities including arthritis, fractures, and other chronic conditions [4–6]. Managing pain in this population is particularly challenging as cognitive impairment interferes with accurate pain assessment [2]. While opioids are commonly prescribed for moderate to severe pain, they carry substantial risks in the elderly population, including respiratory depression, falls, sedation, and confusion [7]. These risks are particularly concerning at the start of therapy, due to lower tolerance and increased overdose susceptibility [8–10]. Some evidence also suggests that opioid exposure may itself be a risk factor for developing dementia in certain age groups [11]. For dementia patients, who often struggle to communicate pain symptoms, the potential for adverse outcomes may be even greater [2].

Given their vulnerability, several studies have examined the incident opioid exposure in this group. Taipale et al. [12] reported an elevated risk of hip fracture among newly opioid-exposed patients (adjusted hazard ratio, aHR; 1.96) in the first 2 months. Hamina et al. [13] observed a higher risk of hospital-treated pneumonia in dementia patients exposed to opioids. More recently, Jensen-Dahm et al. [14] found a substantially increased mortality risk associated with opioid use in dementia patients (aHR, 4.13 within 180 days), with the highest risk observed in the first 14 days (aHR, 10.95).

While these findings underscore the importance of monitoring during early opioid exposure, the use of unexposed patients as a reference group may introduce substantial confounding by indication. Patients who are prescribed medications, such as opioids, often have underlying health issues that make them inherently sicker than those who are not prescribed these treatments, potentially biasing comparisons. To better isolate the short-term risks associated with initiating opioids in patients with dementia, we compared individuals who began opioid therapy after diagnosis (opioid-naïve) with those who had ongoing opioid exposure during the year prior to diagnosis (opioid non-naïve). This approach allowed us to assess short-term mortality risk following opioid initiation, while minimizing confounding introduced by differences in baseline health status. We hypothesized that opioid-naïve patients, those newly exposed post-diagnosis, would experience higher mortality rates due to lower physiological tolerance to opioids and higher sensitivity to their adverse effects. We replicated our findings from a primary cohort of academic hospitals using a larger community healthcare system spanning western US states. In addition, we used a large language model (LLM) to analyze unstructured clinical notes in the primary cohort, enabling detailed characterization of health conditions present at death.

## Methods

### Study design and participants

This retrospective cohort study was primarily conducted at Stanford Health Care Alliance (SHCA), a large academic healthcare system. The study was approved by the Stanford University Institutional Review Board (Protocol 47,644). We replicated our findings using data from Providence Health & Service (PHS), a large, not-for-profit integrated U.S. community healthcare system serving both urban and rural populations across seven states (Alaska, California, Montana, Oregon, New Mexico, Texas, and Washington), with most care sites concentrated in the western US. This replication study was approved by the PHS Institutional Review Board (Protocol STUDY2024000402).

**Fig. 1:**
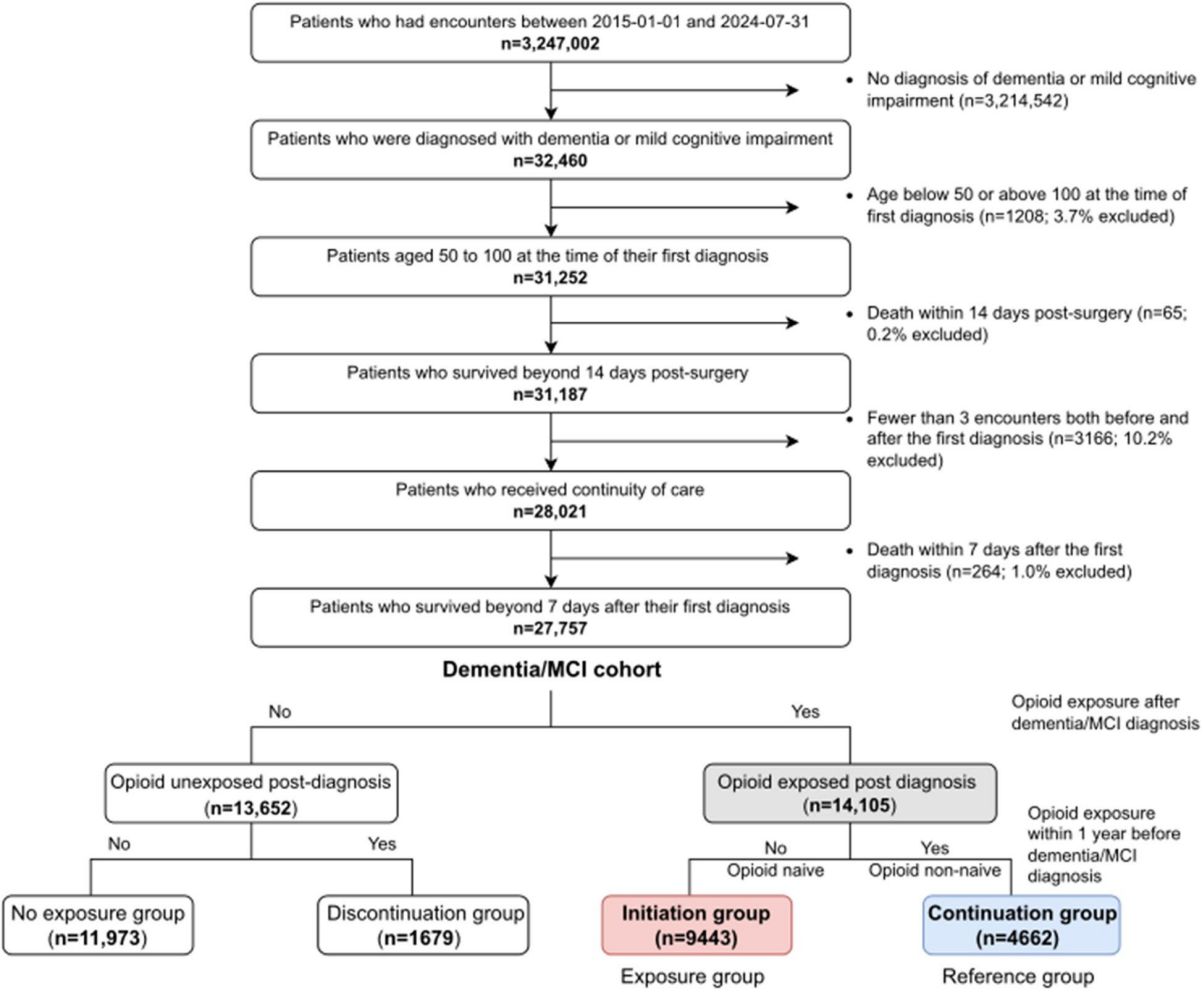
Flowchart of primary cohort and exposure group selection. Descriptive statistics of the dementia/MCI cohort comparing opioid-exposed and unexposed groups are presented in Additional file: Table S8. Descriptive statistics of the opioid-exposed cohort comparing initiation group and continuation group are presented in Table 1 and Additional file: Table S7

Cohort selection of the primary cohort is described in Fig. 1. From individuals with encounters at SHCA between 2015/01/01 and 2024/07/31 (*n* = 3,247,002), we identified those with at least one diagnosis of dementia or mild cognitive impairment (MCI) (*n* = 32,460; Additional file 1: Table S1). We included MCI due to potential overlap with early-stage dementia. We restricted the cohort to individuals aged 50–100 at the time of first diagnosis (*n* = 31,252) and excluded those who died within 14 days of a surgical procedure (*n* = 31,187), to minimize potential confounding from perioperative mortality. This 14-day window aligns with our primary outcome of short-term mortality within 14 days of opioid initiation. Most surgical deaths also occur within the first 7 days, supporting the relevance of this time frame [15]. To ensure continuity of care, we included only individuals with at least three encounters both before and after their first diagnosis and those who survived more than 7 days after their initial dementia/MCI diagnosis (*n* = 27,757).

The same inclusion criteria were applied to the PHS replication cohort during an observation period 2015/01/01–2023/05/31, yielding 207,873 patients (detailed cohort selection is in Additional file 1: Fig. S1).

### Measures

#### Outcome

Our primary outcome was short-term mortality, defined as death within 14 days after the first opioid exposure. We also included follow-up to 180 days as a secondary outcome to observe how mortality patterns evolved over time. Death dates were obtained from the EHR and state’s public records for both SHCA and PHS cohorts. While the EHR provides comprehensive longitudinal health profiles from both structured fields and clinical notes, deaths occurring outside the healthcare system may be missed. Therefore, we incorporated statewide death records to capture out-of-hospital deaths. Although deaths among patients residing outside their state may not be captured, we anticipate minimal impact on our findings given our cohort was restricted to elders receiving continuous care within the system. Patients were followed from the date of opioid initiation until the earliest of death or the end of the 14-day or 180-day follow-up period. Patients were not censored at their last recorded encounter, because all were receiving continuity of care, follow-up periods were relatively short, and death data were supplemented with state records. This approach minimized the likelihood of informative censoring.

#### Exposure

Our exposure of interest was opioid initiation following dementia/MCI onset (Figs. 1, S1, and S2). To isolate the impact of opioid initiation and minimize the confounding by indication, we classified post-diagnosis opioid-exposed patients into two groups based on their opioid exposure in the year preceding dementia/MCI onset: the initiation group (opioid-naïve) and the continuation group (non-naïve). The exposure group (initiation group) comprised individuals who received their first opioid prescription after the onset of dementia/MCI, with no opioid exposure in the preceding year. The reference group (continuation group) included individuals who had been prescribed opioids within one year before their initial dementia or MCI diagnosis and continued receiving them afterward. To ensure comparability and address immortal time bias, both groups were followed from the date of their first post-diagnosis opioid prescription, establishing a consistent time zero. Opioid medications included were buprenorphine, codeine, fentanyl, hydrocodone, hydromorphone, meperidine, methadone, morphine, oxycodone, and tramadol (Additional file: Table S2).

#### Covariates

Patient characteristics and health conditions were collected from EHR prior to the first opioid exposure, including age, race, ethnicity, body mass index (BMI), insurance status, comorbidities, and medication (variable definitions in Additional file: Table S3).

We included comorbidities associated with dementia and mortality, from the two years before first opioid exposure, spanning cardiovascular, respiratory, metabolic, liver, renal, cancer, neurological, mental health, and other conditions (complete list with ICD codes in Additional file: Table S4).

We examined medication exposure within one year before first opioid exposure, focusing on anti-dementia drugs [16], medications in American Geriatrics Society’s (AGS) Beers Criteria [17], and clinician-recommended (JMH) dementia-relevant medications. Medications were grouped into therapeutic/chemical subcategories (complete list with RxNorm codes in Additional file: Table S5).

### Analyses

#### Descriptive analysis

We compared covariates between initiation and continuation groups using chi-squared tests for categorical variables and Welch’s t-test for continuous variables, with Benjamini–Hochberg correction. We also performed descriptive analysis of opioid-exposed versus unexposed patients.

#### Statistical analysis

##### Main analysis

We employed Cox proportional hazards model to estimate hazard ratios (HR) for 14-day mortality, our primary outcome, using lifelines PyPI (v 0.29.0) [18]. To complement the Cox model and examine the time-varying impact of opioid initiation on mortality, we also applied Aalen’s additive hazards model using follow-up data up to 180 days.

Model performance for the Cox model was assessed with concordance index (C-index). C-index ranges from 0.5 to 1, where 1 indicates perfect prediction. For the Aalen model, we used the Integrated Brier Score (IBS) to evaluate prediction accuracy, where lower values indicate better performance. We used the C-index for Cox to assess discrimination and the IBS for Aalen to evaluate overall prediction error over time, reflecting each model’s assumptions and outputs.

We identified stabilization points where the smoothed first derivative (10-day rolling mean) of cumulative hazard remained below 5% of its maximum for 10 consecutive days. Variables with <5% cases were excluded, and missing values were imputed using medians. Analysis was limited to 180 days, based on the assumption that the impact of opioid initiation diminishes over time and confounding from other factors increases.

##### Subgroup, sensitivity, post-hoc supplementary analysis

For sensitivity analyses, we compared the initiation group with the long-term continuation group (≥90 days pre-diagnosis opioid exposure) and tested the proportional hazard assumption using Schoenfeld residuals and the Kolmogorov-type supremum test. To assess whether excess risk was concentrated early, we also stratified the 14-day follow-up into 0–7 and 8–14 day intervals and fitted separate Cox models for each period. We conducted subgroup analyses by opioid strength (categorized as weak or strong based on the WHO guidelines for the pharmacological management of cancer pain) [19], dementia/MCI status (not mutually exclusive), and medication order mode (inpatient vs. outpatient). Based on analysis of unstructured notes, we examined pneumonia’s role by analyzing patients who had a pneumonia diagnosis within 7 days prior to opioid initiation and by comparing pneumonia risk between initiation and continuation groups among those without prior pneumonia.

#### Identification of potential reasons for short-term mortality

To examine causes of death within 14 days of first opioid exposure, we securely processed clinical notes from the three days preceding death using GPT-3.5 Turbo to identify the top three contributing conditions in the primary cohort (Additional file 1: Table S6) [20]. This was done to uncover clinical correlations not readily apparent in structured data alone. To evaluate the accuracy of GPT outputs, we reviewed 50 randomly selected notes for hallucination-related errors and compared another 50 notes with explicit clinician-documented causes of death for in-hospital deaths. Health conditions were categorized into predefined categories using GPT-4o with clinician (JMH) review of mappings. Multiple category assignments were allowed per patient.

### Replication study

Variables were collected using similar methods as the primary cohort, adapted for different data models. The same analyses were performed, except for GPT analysis.

## Results

Among 27,757 individuals with dementia/MCI, 14,105 (50.8%) received opioids post-diagnosis: 9443 initiation and 4662 continuation groups (Fig. 1). The opioid-exposed cohort was predominantly female (56.0%), White (64.2%), non-Hispanic (88.0%), Medicaid/Medicare insured (53.3%), with normal BMI (42.0%), and a median age of 81 years (IQR: 73–87). Mortality rates were 4.1% at 14 days, 8.9% at 60 days, and 14.2% at 180 days (Fig. 1 and Table 1, S7, S8).

**Table 1:** Descriptive statistics of the primary analytic cohort

	Missing	Overall (*n* = 14,105)	Initiation group (*n* = 9443)	Continuation group (*n* = 4662)	*P*
Dementia category					
Mild cognitive impairment, *n* (%)		5213 (37.0)	3182 (33.7)	2031 (43.6)	< 0.001
Alzheimer’s disease, *n* (%)		3160 (22.4)	2411 (25.5)	749 (16.1)	< 0.001
Vascular dementia, *n* (%)		1319 (9.3)	881 (9.3)	438 (9.4)	0.93
Other/unspecified dementia, *n* (%)		10,637 (75.4)	7390 (78.3)	3247 (69.6)	< 0.001
Patient characteristics					
Sex	1				1.000
Female		7904 (56.0)	5292 (56.0)	2612 (56.0)	
Male		6202 (44.0)	4152 (44.0)	2050 (44.0)	
Age at diagnosis, median (Q1,Q3)	0	81.0 (73.0,87.0)	81.0 (74.0,87.0)	79.0 (71.0,86.0)	< 0.001
Age group, *n* (%)	0				< 0.001
50–59		695 (4.9)	398 (4.2)	297 (6.4)	
60–69		1720 (12.2)	986 (10.4)	734 (15.7)	
70–79		4080 (28.9)	2643 (28.0)	1437 (30.8)	
80–89		5499 (39.0)	3856 (40.8)	1643 (35.2)	
90–100		2113 (15.0)	1561 (16.5)	552 (11.8)	
Race, *n*(%)	57				< 0.001
White		9021 (64.2)	6007 (63.8)	3014 (65.0)	
Black		2019 (14.4)	1433 (15.2)	586 (12.6)	
Asian		725 (5.2)	422 (4.5)	303 (6.5)	
Native American/Hawaiian/Pacific Islander		117 (0.8)	73 (0.8)	44 (0.9)	
Unknown/refused		265 (1.9)	209 (2.2)	56 (1.2)	
Other		1903 (13.5)	1266 (13.5)	637 (13.7)	
Ethnicity, *n* (%)	13				< 0.001
Hispanic/Latino		1326 (9.4)	835 (8.9)	491 (10.5)	
Non-Hispanic/Latino		12,402 (88.0)	8326 (88.2)	4076 (87.5)	
Unknown/refused		366 (2.6)	274 (2.9)	92 (2.0)	
Insurance, *n* (%)	226				0.78
Medicare/Medicaid		7402 (53.3)	4932 (53.2)	2470 (53.6)	
Private		4481 (32.3)	3013 (32.5)	1468 (31.9)	
Other		1998 (14.4)	1332 (14.4)	666 (14.5)	
BMI, median (Q1,Q3)	914	25.4 (22.3,28.9)	25.1 (22.1,28.6)	25.8 (22.6,29.6)	< 0.001
BMI category, *n* (%)	1158				< 0.001
Under weight		624 (4.8)	426 (5.0)	198 (4.5)	
Normal weight		5436 (42.0)	3717 (43.5)	1719 (39.1)	
Over weight		4451 (34.4)	2913 (34.1)	1538 (34.9)	
Obese		1812 (14.0)	1127 (13.2)	685 (15.6)	
Severely obese		626 (4.8)	365 (4.3)	261 (5.9)	
Death outcome					
Death, *n* (%)	0	6104 (43.3)	4140 (43.8)	1964 (42.1)	0.06
Days to death from first opioid exposure, mean (SD)	8004	837.4 (910.6)	837.7 (913.9)	836.8 (904.0)	0.97
Death within 14 days after first opioid exposure, *n* (%)	0	572 (4.1)	451 (4.8)	121 (2.6)	< 0.001
Death within 60 days after first opioid exposure, *n* (%)	0	1261 (8.9)	882 (9.3)	379 (8.1)	0.02
Death within 180 days after first opioid exposure, *n* (%)	0	1997 (14.2)	1379 (14.6)	618 (13.3)	0.03

Categorical variables were compared using the chi-squared test. Continuous variables were evaluated using Welch’s *t*-test. The Benjamini–Hochberg method was used to correct for multiple testing errors. All analyses were conducted using the PyPI package tableone (v.0.9.1) [29]. Variables that contained cells with fewer than 20 cases were dropped to protect patient privacy. Variables are defined in Additional file 1: Table S3

Compared to the continuation group, patients in the initiation group were older, had more Asian and fewer Black/Hispanic patients, less obesity, higher dementia, and lower MCI prevalence (*P*<0.001, Table 1). They had fewer comorbidities (33/34 conditions) and medications (43/45; *P*<0.05). Mortality was higher in the initiation group at 14 days (4.8% vs. 2.6%, *P*<0.001), 60 days (9.3% vs. 8.1%, *P*<0.05), and 180 days (14.6% vs. 13.3%, *P*<0.05; Additional file 1: Tables S7).

The replication cohort is described in Additional file 1: Table S9. Of 208,306 individuals with dementia or MCI, 113,343 received opioids post-diagnosis (77,168 initiation group and 36,175 continuation group). Compared to the primary cohort, the replication cohort had more dementia (85.8% vs. 78.4%; *P*<0.0001) and fewer MCI diagnoses (26.9% vs. 36.9%; *P*<0.0001), higher proportions of female (60.2% vs. 56.0%; *P*<0.0001), White (82.2% vs. 64.2%; *P*<0.0001), non-Hispanic (91.2% vs. 88%; *P*<0.0001), and Medicare/Medicaid (92.7% vs. 53.3%; *P*<0.0001) patients. Overall mortality rates were higher in the replication cohort compared to the primary cohort (14-day: 5.6% vs. 4.0%, 60-day: 10.3% vs. 9.1%, 180-day: 14.9%; *P*<0.0001). The patterns observed in the primary cohort, differences between initiation and continuation groups in patient characteristics, comorbidities, medication exposures, and mortality, were similarly observed in the replication cohort.

**Fig. 2:**
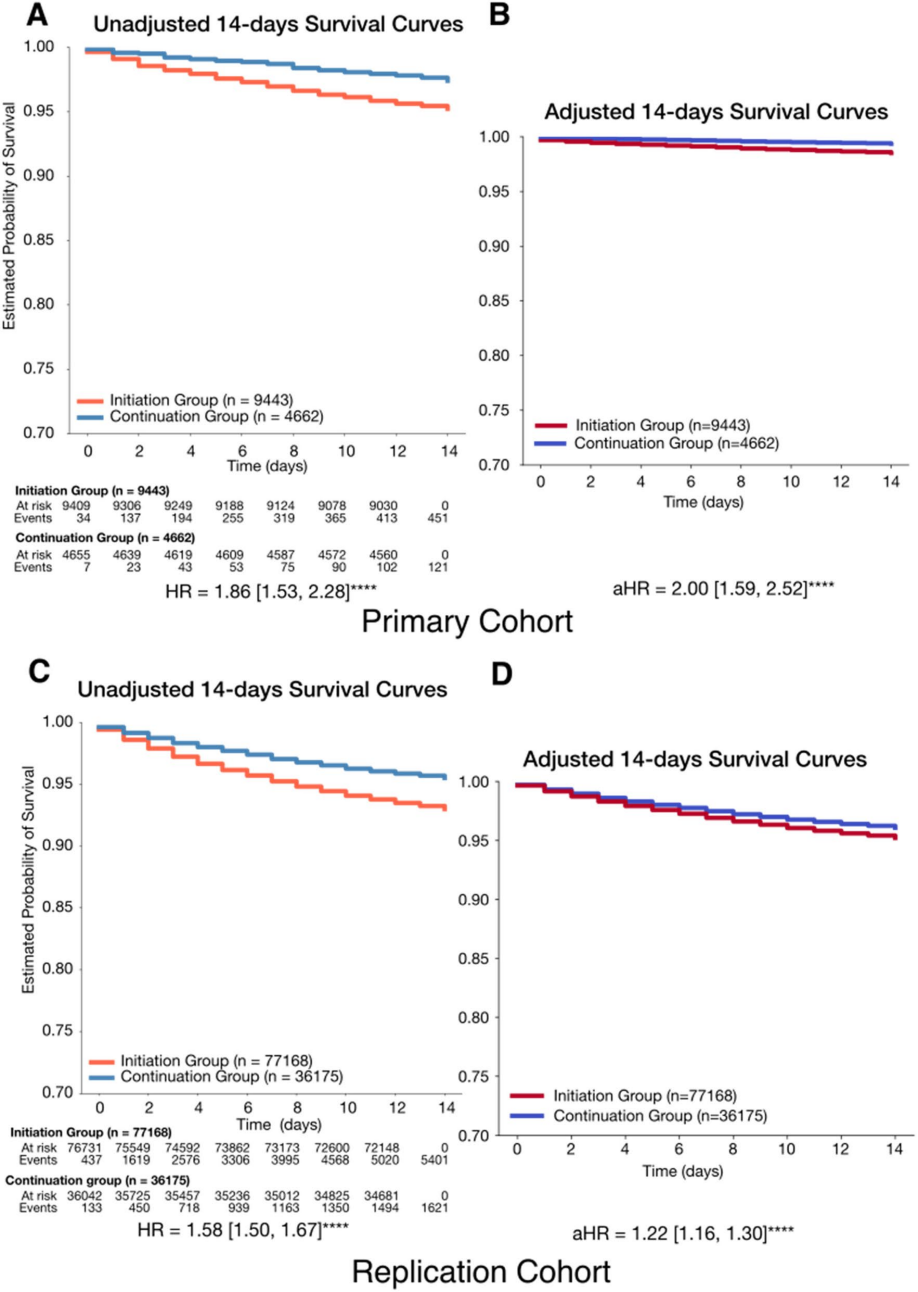
Fourteen-day survival probability curves comparing initiation group and continuation group. **A** Unadjusted 14-day survival probability curves comparing initiation group and continuation group. **B** Adjusted 14-day survival probability curves comparing initiation group and continuation group. Initiation group (*n* = 9443) had a lower likelihood of survival during the 14-day follow-up period, with an adjusted hazard ratio (aHR) of 2.00 (1.55, 2.47) (*P* < 0.0001). The model achieved a concordance index of 0.78, indicating good predictive performance. **C** Unadjusted 14-day survival probability curves comparing initiation group and continuation group. **D** Adjusted 14-day survival probability curves comparing initiation group and continuation group. Initiation group (*n* = 77,168) had a lower likelihood of survival during the 14-day follow-up period, with an adjusted hazard ratio (aHR) of 1.22 (1.16, 1.30) (*P* < 0.0001). The model achieved a concordance index of 0.85, indicating good predictive performance. Hazard ratios were computed using a Cox proportional hazards model, adjusted for age at diagnosis, race, ethnicity, body mass index (BMI), insurance status, comorbidities, and medication exposures. Complete lists of comorbidities and medications can be found in Tables S4 and S5. Variables with cases fewer than < 5% of analytic cohort were excluded from the analysis. Significance levels: + : *P* < 0.1, *: *P* < 0.05, **: *P* < 0.01, ***: *P* < 0.001, ****: *P* < 0.0001

**Fig. 3:**
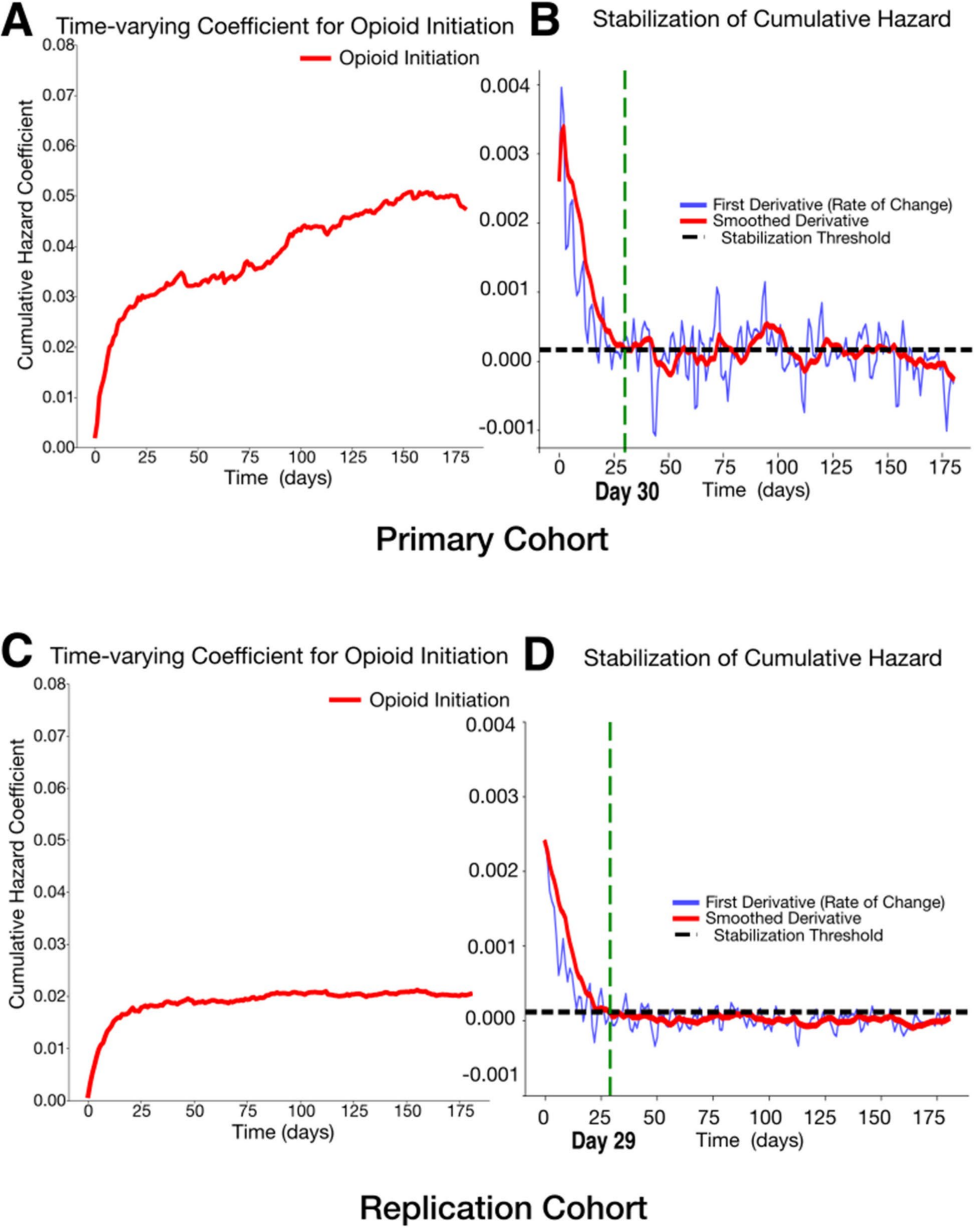
Time-varying effects of opioid initiation on cumulative hazard and stabilization. **A** The cumulative hazard coefficient for opioid initiation over time in the primary cohort (Integrated Brier Score (IBS): 0.08, IBS ranges from 0 to 1. IBS of 0 means perfect accuracy). **B** The first derivative (rate of change) of the cumulative hazard (blue) and its smoothed version (red), with the stabilization threshold (black dashed line) in the primary cohort. **C** The cumulative hazard coefficient for opioid initiation over time in the replication cohort (IBS: 0.09). **D** The first derivative (rate of change) of the cumulative hazard (blue) and its smoothed version (red), with the stabilization threshold (black dashed line) in the replication cohort. The green dashed line marks the estimated stabilization point (day 30 for the primary cohort and day 29 for the replication cohort), indicating when the cumulative hazard change stabilizes

In the primary cohort, the initiation group showed significantly higher 14-day mortality risk, with an unadjusted HR of 1.86 (1.53, 2.28) increasing to 2.00 (1.59, 2.52) after adjustment (*P*<0.0001; C-index: 0.78; Fig. 2). In the replication cohort, the adjusted HR was 1.22 (1.16–1.30) (*P*<0.0001), compared to an unadjusted HR of 1.59 (1.50–1.68), with a C-index of 0.85 (Fig. 2). Over 180 days, both cohorts showed consistently higher hazards in the initiation group (Additional file 1: Fig. S3), with time-varying cumulative hazard stabilizing around day 30 (Fig. 3, IBS; primary: 0.08, replication: 0.09). The cumulative hazard continued to rise in the primary cohort but plateaued in the replication cohort (Fig. 3).

Sensitivity analyses comparing initiation group to long-term continuation group showed similar survival trends and hazard ratios consistent with the main analysis in both cohorts (Additional file 1: Fig. S4). Accounting for variables that violated the proportional hazards assumption had a minimal impact on the results. In a time-stratified analysis, the mortality risk among initiators was highest in the first 7 days (aHR = 2.53 (1.85–3.48); *P*<0.0001) and remained elevated but attenuated during days 8 to 14 (aHR = 1.42 (1.01–2.00); *P* = 0.04), suggesting a critical early risk period.

Subgroup analyses consistently showed higher mortality risk for the initiation group, with some differences between cohorts. In the primary cohort, mortality risk was higher with stronger opioids, in patients with dementia, and among inpatients. The replication cohort showed smaller hazard ratios overall, with larger hazard ratios in the MCI and inpatient groups, though dementia and outpatient groups had higher absolute mortality (Additional file 1: Figs. S5, S6, and S7).

**Fig. 4:**
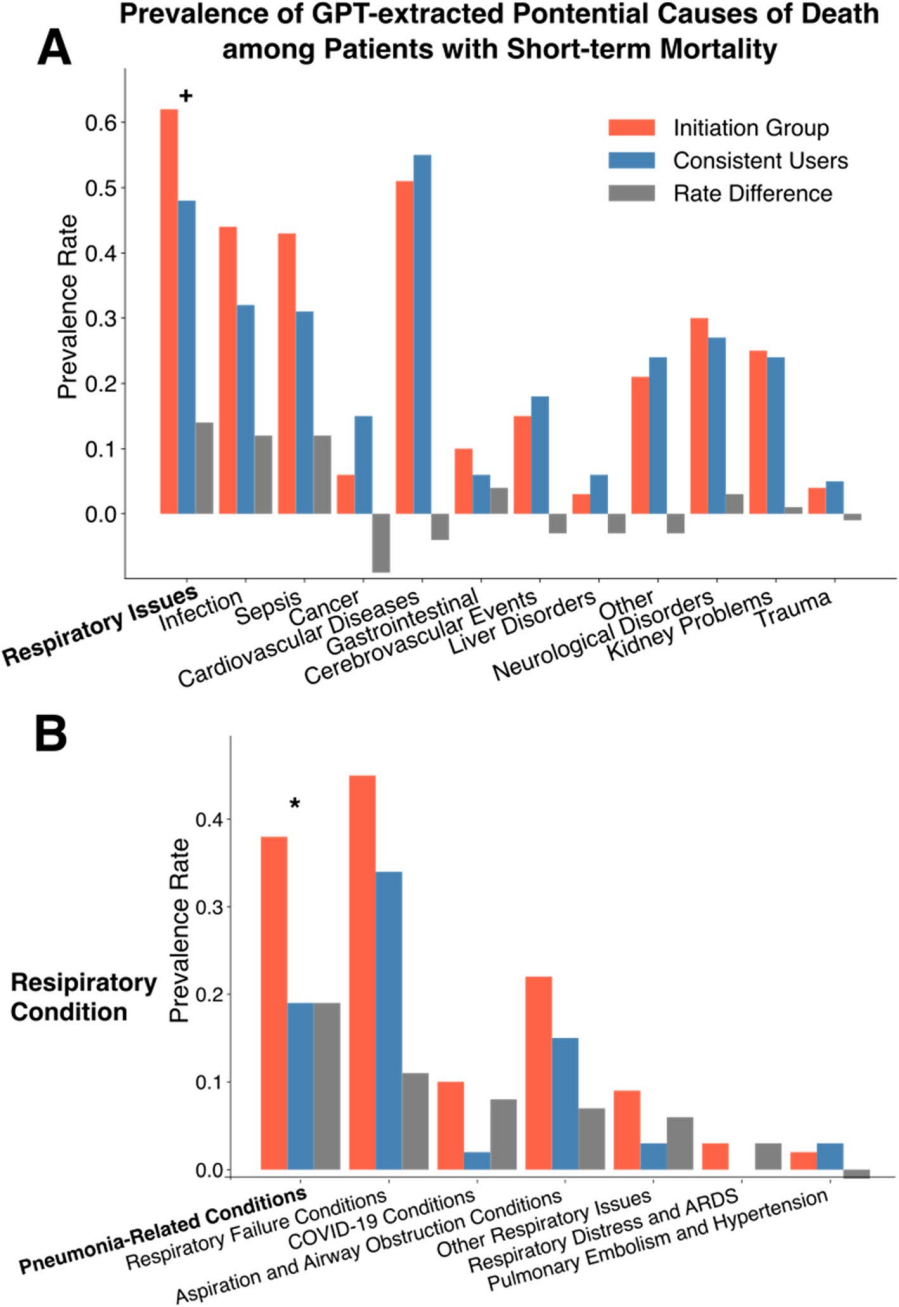
Bar plot of prevalence rate difference in GPT-extracted potential causes of death between initiation and continuation groups in primary cohort. Plot **A** shows the difference in prevalence rates of GPT-extracted potential causes of death between initiation and continuation groups, shown as a bar plot. Plot **B** shows the difference in prevalence rate focusing on specific respiratory-related conditions. We collected clinical notes mentioning death among individuals who passed away within 14 days after opioid initiation (438 notes for 179 patients in initiation group and 138 notes for 62 patients in continuation group). These notes were processed through secure GPT-3.5 Turbo to identify potential causes of death. The prompt used for this analysis is provided in Additional file 1: Table S6. Among 50 randomly selected notes, no instances of hallucination were detected. If a note did not contain information about the health condition at the time of death, GPT-3.5 Turbo outputted “NA.” When a primary cause of death was specified, it was listed as the first cause; if unspecified, GPT-3.5 Turbo identified the primary health concern mentioned in the note at the time of death. We utilized GPT-4o to categorize these potential causes into predefined health condition categories. If a cause fit into multiple categories, it was assigned to all relevant categories. A clinician (JMH) reviewed the cause-category mappings to ensure accuracy, and adjustments were made based on their feedback. Significance levels: + : *P* < 0.1, *: *P* < 0.05, **: *P* < 0.01, ***: *P* < 0.001, ****: *P* < 0.0001

Within 14 days of their first opioid exposure post diagnosis, 450 patients in the initiation group and 121 patients in the continuation group died (Table 1). We processed 438 notes for 179 patients in the initiation group and 138 notes for 62 patients in the continuation group. No hallucinations were detected in a random 50 notes. In another random 50 notes with a specified principal diagnosis at death, 49 matched the specified principal diagnosis category (46 exact, 3 category match), and one partially matched (Additional file 1: Table S10). Figure 4 shows that respiratory-related conditions exhibited the largest difference in prevalence between the groups, occurring in 62% of the initiation group compared to 48% of the continuation group (*P*=0.09). A more detailed breakdown of respiratory issues revealed that respiratory failure was the most common condition in both groups. However, pneumonia-related conditions were particularly high among the initiation group, with a 19% higher prevalence (*P*=0.04).

Opioid initiation was associated with a higher mortality risk in patients with pre-existing pneumonia in the primary cohort (aHR = 2.45 (1.51, 3.96), *P*<0.001) but not in the replication cohort. For patients without pre-existing pneumonia, the replication cohort showed a slight but significant increase in pneumonia risk (aHR = 1.22 (1.08, 1.38), *P*<0.01), while the primary cohort did not (Additional file 1: Fig. S8).

## Discussion

Initiating opioids in opioid-naïve patients with dementia or MCI was associated with increased 14-day mortality compared to those with prior opioid exposure. This association was consistent across both an academic medical center and a larger community healthcare system, persisting through sensitivity and subgroup analyses. The heightened risk associated with opioid initiation stabilized by day 30 in both cohorts.

Our study is the first to compare short-term mortality between patients who newly initiated opioids after dementia diagnosis and those with prior and ongoing opioid exposure, reducing confounding by indication. A recent study from Denmark reported an 11-fold increase in 14-day mortality risk associated with opioid exposure among patients with dementia. Their analysis compared opioid-exposed to unexposed patients using a 1-year washout period before dementia diagnosis [14]. Instead, we focused within the opioid-exposed group, assuming underlying health conditions would be similar between the exposure group (initiation group) and reference group (continuation group). In our study, the continuation group even had higher rates of comorbidities and medication exposure, and the initiation group still showed elevated mortality risk.

We found elevated 14-day mortality risk among the initiation group, with this association persisting across distinctly different healthcare settings. Both the Northern California academic medical center and the western US community hospital system showed increased mortality risk, as confirmed through comprehensive sensitivity and subgroup analyses. The consistency of this finding is particularly noteworthy given the distinct differences in healthcare delivery models and patient populations between these institutions. The replication cohort, serving both rural and urban communities through a not-for-profit system, had a predominantly government-insured population (93%) compared to the academic center’s more diverse payer mix (52%), reflecting substantial variations in patient characteristics and socioeconomic factors. Previous research has established that such institutional and demographic differences can significantly influence clinical outcomes [21–23]. Interestingly, the same covariate adjustments increased the hazard ratio in the primary cohort but reduced it in the replication cohort. These findings strengthen the generalizability of our results while highlighting the need for healthcare system-specific approaches to risk mitigation. Given the observed differences in patient populations, institutional characteristics, magnitude of risk, and subgroup analyses, we recommend developing tailored intervention strategies that account for each system’s unique contextual factors, resources, and constraints while maintaining vigilance regarding opioid-associated mortality risk.

After 30 days, the mortality risk associated with opioid initiation began to stabilize across settings. While previous studies have demonstrated elevated risks of opioid-related adverse effects during the initial phase of treatment in elderly populations [12, 14, 24], these comparisons were between opioid-exposed and unexposed patients and lacked a specific timeframe. Our study took a novel approach by comparing the initiation group to the continuation group within the exposed group, identifying a specific 30-day window of heightened risk, providing clinicians with an actionable timeframe for intervention. This 30-day period suggests a timeframe for close monitoring of patients newly initiating opioid therapy, potentially through weekly follow-up for adverse events and increased patient and caregiver education about monitoring opioid-related adverse effects.

Pneumonia emerged as a significant factor in the association between short-term mortality and opioid initiation. In the primary cohort, we found a particularly high prevalence of pneumonia among the initiation group as a potential cause of early mortality, based on GPT-extracted causes of death. This prompted us to investigate whether the association reflected pneumonia resulting from opioid exposure, as suggested by previous studies [13, 24, 25], or preexisting conditions. Our supplementary analyses revealed contrasting patterns between cohorts. In the primary cohort, patients with preexisting pneumonia showed a 2.38-fold increase in short-term mortality after opioid initiation, while opioid initiation did not increase subsequent pneumonia risk among those without preexisting pneumonia. The replication cohort showed an opposite pattern: opioid initiation was associated with a 1.2-fold increased risk of subsequent pneumonia, but not with increased mortality among patients with preexisting pneumonia. These contrasting findings likely reflect the interaction between opioid-induced respiratory depression and pneumonia, manifesting differently across healthcare settings: exacerbating existing pneumonia in the primary cohort while increasing susceptibility to new infections in the replication cohort [10, 26, 27]. These differences highlight the need for site-specific risk mitigation strategies. Our analysis did not examine specific opioid dose–response relationships in relation to adverse events, which may explain the disparate findings across healthcare settings. Nonetheless, our findings emphasize the importance of monitoring for pneumonia among patients with dementia who are newly prescribed opioids, and also carefully considering the risks and benefits of opioid prescribing among opioid-naive patients with co-morbid dementia and pneumonia.

The integration of LLM-based clinical note analysis with traditional structured data analysis strengthened our research methodology. While structured data analysis established the mortality patterns, LLM analysis of clinical notes identified pneumonia as a significant factor, enabling targeted supplementary analyses that revealed complex relationships between pneumonia timing, opioid initiation, and mortality across healthcare settings. This complementary approach demonstrates how unstructured clinical notes can provide crucial context often missed in structured data alone, allowing us to generate and test hypotheses about complex clinical outcomes.

Our study has several important limitations. First, while we obtained death dates from both EHR and state death records, we could not access clinical notes from outside facilities, potentially limiting our understanding of health status at death for patients who died in other healthcare settings. Second, we could only verify medication administration for inpatient care: our outpatient data only captured prescriptions and dispensing records, and not actual adherence. This introduces potential exposure misclassification. However, such misclassification is likely nondifferential between groups and would bias results toward the null, suggesting that the observed association may underestimate the true short-term risk of opioid initiation. Third, our identification of MCI and dementia relied on a single diagnostic code. Although this approach has been shown to yield high specificity, it may undercapture milder or less consistently documented cases due to lower sensitivity [28]. As our study focused on patients with a documented diagnosis, the impact of this potential misclassification on internal comparisons is likely limited. Fourth, our use of GPT for analyzing clinical notes was limited to the primary cohort. Fifth, the higher mortality risk among the initiation group could be partially attributed to acute severe conditions necessitating first-time opioid exposure, such as traumatic injuries or accidents. However, our GPT analysis of clinical notes from the primary cohort revealed that such acute traumatic events were relatively uncommon causes of death, suggesting that this potential confounding factor may not fully explain the observed mortality difference. We also observed differences in subgroup patterns across cohorts, including a reversal in the association by MCI versus dementia diagnosis. These differences may reflect variation in diagnostic coding practices and care delivery contexts across health systems. While we explored potential explanations, further investigation was beyond the scope of this study, and our limited ability to account for system-level factors raised concerns about introducing speculative or potentially misleading interpretations. For this reason, we intentionally did not pursue additional stratified analyses or modeling of these differences. Finally, while our study focused on risk estimation within a short follow-up window, future research aiming to estimate causal effects, particularly over extended follow-up periods, may benefit from applying formal causal inference methods such as inverse probability weighting or G computation.

## Conclusions

This study demonstrates significantly elevated short-term mortality risk associated with initiating opioid therapy in patients with dementia, particularly within the first 14 days and stabilizing by day 30. The consistency of these findings across distinctly different healthcare settings, despite varying magnitudes of risks, strengthens their generalizability while highlighting the need for setting-specific risk mitigation strategies. The heightened vulnerability of opioid-naïve patients to adverse effects emphasizes the importance of careful risk assessment and close monitoring when initiating opioid therapy in this vulnerable population.

## Supplementary Information

Additional File 1: Tables S1-S10 and Figures S1-S7. Table S1 - ICD-10 codes and search criteria of dementia and its subcategories. Table S2 - RxNorm codes of opioid ingredient. Table S3 - Variable definition. Table S4 - ICD-10 codes and inclusion criteria of comorbidities. Table S5 - RxNorm codes, categories, and inclusion criteria of medications. Table S6 - Secure GPT-3.5 Turbo prompt for potential causes of death identification. Table S7 - Descriptive statistics of opioid-exposed patients, comparing initiation group and continuation group in primary cohort on comorbidities and medication exposure. Table S8 - Descriptive statistics of the dementia/mild cognitive impairment cohort based on post-diagnosis opioid exposure status. Table S9 - Descriptive statistics of opioid-exposed patients, comparing initiation group and continuation group in replication cohort. Table S10 - Notes, GPT Responses, and Health Condition Categories of Mismatches Between Specified Principal Diagnoses at the Time of Death and GPT Responses. Figure S1 - Flowchart of cohort and exposure group selection in the replication cohort. Figure S2 - Study design and definition of exposure groups. Figure S3 - 180-day survival probability curves comparing initiation group and continuation group in primary and replication cohorts. Figure S4 - Sensitivity analysis comparing first-time opioid users to long-term continuation group in primary and replication cohort. Figure S5 - Strong opioid-specific and weak-opioid specific 14-day survival probability curve comparing initiation group and continuation group in primary and replication cohort. Figure S6: MCI-specific and dementia-specific 14-day survival probability curve comparing initiation group and continuation group in primary and replication cohort. Figure S7 - Inpatient-specific and outpatient-specific 14-day survival probability curve comparing initiation group and continuation group in primary and replication cohort. Figure S8 - Post-hoc supplementary analysis of short-term mortality associated with opioid initiation in patients with preexisting pneumonia and pneumonia development risk following opioid initiation in patients without preexisting pneumonia in primary and replication cohort.

## Data Availability

Data cannot be shared for ethical/privacy reasons. However, all clinical logic has been transparently shared. The results have been aggregated and reported in this manuscript to the fullest extent possible while adhering to legal requirements for protecting personal health information. The code is available in the GitHub repository: https://github.com/su-boussard-lab/dementia-opioid-initiation/tree/main.

## Abbreviations

aHR: Adjusted hazard ratio
BMI: Body mass index
CI: Confidence interval
GPT: Generative pre-trained transformer
HR: Hazard ratio
IQR: Interquartile range
LLM: Large language model
MCI: Mild cognitive impairment
US: United States
